# Findings from the development of a novel course of both group and individual Alexander Technique lessons for neck, hip, and knee pain: a mixed-methods study

**DOI:** 10.3399/BJGPO.2024.0295

**Published:** 2025-12-19

**Authors:** Joseph Little, Adam Geraghty, Carolyn Nicholls, Paul Little

**Affiliations:** 1 Primary Care Research Centre, Faculty of Medicine, University of Southampton, Southampton, UK; 2 Brighton Alexander Technique College, Brighton, UK

**Keywords:** pain, neck, hip, knee

## Abstract

**Background:**

Musculoskeletal pain, often affecting multiple sites — including neck, hip, and knee — is common, with limited treatment options. Novel treatments are needed to support self-management, preferably addressing multiple pain sites.

**Aim:**

To develop and explore the acceptability of a short, mixed course of individual (one-to-one) and group lessons in the Alexander Technique (AT), which addresses dysfunctional use of the musculoskeletal system.

**Design & setting:**

A single-centre, mixed-methods study of patients with chronic or recurrent neck, hip, or knee pain from four general practices.

**Method:**

Preliminary development of a course of 10 AT lessons (six group, four individual) took place with a group of AT teachers. Semi-structured interviews of participants were undertaken, which were analysed using inductive thematic analysis. Descriptive pre-post analysis of quantitative scales were used to assess improvement (Numerical pain scale [NRS]; modified Roland–Morris Disability Questionnaire (RMDQ); enablement (modified Patient Enablement Instrument used in the ATEAM trial); and global improvement (Health Transition scale).

**Results:**

Twenty-three participants were included; 18 were interviewed. Commonly, participants found the mixture of group and individual lessons helpful, including helping multiple pain sites, and the mix of different problems enhanced learning. There was moderate improvement in standard quantitative measures over 12 weeks (NRS from 5.15 to 3.85; modified RMDQ 8.26 to 5.7) but with more substantial improvement in enablement and global improvement. Those who perceived underlying structural damage to the knee reported little benefit.

**Conclusion:**

People with chronic or recurrent neck, hip, or knee pain found a course of mixed group and individual lessons in AT helpful in managing their pain, but not those with severe knee problems. Whether standard quantitative measures provide the best measures of effectiveness requires exploration.

## How this fits in

Back, neck, knee, and hip pain, commonly affecting several sites, are the commonest causes of chronic or recurrent pain, and there are limited treatment options and evidence for interventions. Individual lessons in Alexander Technique (AT), which addresses dysfunctional use of the musculoskeletal system, is effective for back and neck pain. The cost of a full course of individual lessons is high, so exploring the acceptability and impact of shorter courses and including group lessons is warranted. Participants with chronic or recurrent neck, hip, or knee pain reported acceptability of a short course of mixed group and individual lessons in AT. Most participants reported benefit in managing their pain, but not those with severe knee problems.

## Introduction

Back, neck, knee, and hip pain, usually owing to osteoarthritis (OA), are the commonest causes of chronic pain and have major impacts on health status and quality of life (QOL). ^1–5^ The impact increases with the number of joints affected,^1,4^ and at least 10% of the population consult primary care.^6^ There are limited treatment options; the National Institute for Health and Care Excellence (NICE) guidance for OA^7^ recommends maintaining activity and exercise, and weight loss if overweight and considering local hot or cold manipulation and stretching, transcutaneous electrical nerve stimulation (TENS), and modifications to footwear. For neck pain, guidelines from NICE Clinical Knowledge Summaries (CKS)^8^ advise analgesics, activity, considering muscle relaxants, the use of pillows, and referrals if not responding to simple advice and medication. For back pain, NICE recommends group-based elements for cost-effective management,^9^ and there is moderate evidence to support groups for other conditions.^10^ Group elements probably provide reinforcement, encouragement, empowerment to change behaviour, solidarity in the common struggle with illness, and the opportunity to share tips and advice,^11–14^ albeit interpersonal issues can sometimes lessen the value of groups.^15^

Thus, novel treatments are needed to support patients with musculoskeletal pain, preferably those that can sensitively incorporate group elements, and those that address multiple joints and sites that would not only be patient centred, but also could potentially have a bigger impact on the ability to manage pain and functioning.

Lessons in the Alexander Technique (AT) could be a promising option. AT is designed to develop lifelong skills for self-care in avoiding poor habits affecting neuromuscular coordination and postural tone, paying particular attention to release of unwanted tension in the head, neck, and spine, which is guided by verbal instruction and hand contact from the teacher, and spending time between lessons practising and applying the technique. AT was found to be both effective and cost-effective for back pain (Medical Research Council [MRC]-funded Alexander Technique lessons, exercise, and massage [ATEAM] trial),^16,17^ and effective for neck pain^18^ (the ATLAS trial) but not cost-effective owing to the cost of 20 lessons.^19^ For knee and hip pain, poor joint coordination and posture is related to ongoing pain and poor prognosis^20–24^ and AT improved the coordination of the knee joint (reducing medial co-contraction) with clinically important changes in pain and function (Western Ontario and McMaster Universities Osteoarthritis Index [WOMAC] score).^23^ Thus although there was insufficient evidence to include shoulder pain, fibromyalgia, or widespread pain, for some of the common musculoskeletal complaints there is sufficient promising evidence to use AT.

Including some group sessions would substantially increase cost-effectiveness and preliminary evidence from small studies suggests that 10 group AT sessions may work for chronic neck pain,^25^ reduce pain and/or tension in music students, and improve functional reach and balance in older women.^26–29^ A course for back pain (four individual lessons, six group, with groups sizes varying between four and six) was feasible and with preliminary evidence of effectiveness (*n* = 40).^30^ The current study aimed to develop and assess the feasibility and perceived effectiveness of a similar course for neck, hip, or knee pain.

## Method

### Overall study design

This was a mixed-methods study.

### Development and structure of the intervention

The structure of the intervention, the curriculum, and materials used as a starting point the course curriculum for patients with chronic low back pain (also see Template for Intervention Description and Replication [TIDieR] checklist in Supplementary file, Appendix).^30^ Materials were developed by CN in consultation with a peer group of experienced AT teachers, most of whom had taken part in previous trials.^16,17,25^ CN consulted with three senior teachers over a 4-month period, with three in person meetings and ongoing email exchanges of ideas. The whole programme was reviewed several times before completion. Summary learning points and MP3 talks to explain aspects of the technique for home practice of key skills — such as using the semi-supine position — were also developed. Participants attended a course of 10 lessons over approximately 10–12 weeks. Of these, four lessons were one-on-one with their AT teacher with the remaining six lessons being in a small group of 4–6 participants. The groups sessions included some hands-on work with each participant, to provide individualised feedback on body use, during which the teacher explained to the group what they were doing. Each participant was also given a book explaining AT to read in their own time (*Body*, *Breath and Being*).^^24^^ *Body Breath and Being* provides an introduction to help participants understand the technique, dealing with popular myths about ‘posture’, and explaining how poor body use develops, dealing with how to be aware of and inhibit the habitual responses that lead to bad body use and pain, and advising practising active rest (the ‘semi-supine’ position). Individual chapters deal with particular activities (for example, breathing, sitting, walking) and there are suggested activities at the end of each chapter to help practise the technique. Participants were asked to read specific chapters that related to the topics covered in specific group sessions. These chapters enlarged on and enhanced the lesson material. Participants were emailed the day before each group lesson with reminders of time and place and link to relevant chapters. After the group lessons they were emailed with reminders of relevant chapters. Participants were given a record sheet to note their semi-supine (active rest) practice daily.

### Setting and participants

The aim was to include people with chronic or recurrent neck, hip, or knee pain. General practices wrote to a random sample (to ensure a representative sample was invited) of patients who had seen the GP for neck, hip, or knee pain during the past 5 years. Potential participants were screened for eligibility by the trial manager and offered a place on the next available group course.

### Participant inclusion criteria

The inclusion criteria were as follows: aged ≥18 years; ability to understand English (since outcomes were validated in English); chronic or recurrent neck, hip, or knee pain (at least one previous episode recorded on GP electronic records and a current episode at least 3 weeks in duration); and Numerical Rating Scale (NRS) score of ≥4 out of 10.

### Participant exclusion criteria

The exclusion criteria were as follows: previous lessons in AT; unable to reliably answer outcome questions (for example, severe and unstable mental illness, dementia or learning difficulty); unable to sit down owing to pain (prevents elements of AT practice); pregnancy; current nerve root pain below the knee (sciatica); previous spinal surgery or planned major surgery; pending litigation for back pain; terminal illness; and any ‘red-flag’ criteria suggesting sinister pathology.

### Measures and outcomes

Near the end of their course participants were also asked to take part in semi-structured qualitative telephone interviews about their experience of AT and of learning in a group format. Open-ended prompts were used and adapted as the interviews progressed where new issues were identified. Interviews were transcribed verbatim before analysis.

All participants completed a questionnaire at baseline and at 3 months (final follow-up) including basic demographic information; NRS; health-related quality of life (EQ-5D);^31^ a modified version of the RMDQ (so that the questionnaire did not just refer to back pain, but all pain sites);^32^ days in pain and days interference with activity over past week;^33^ overall improvement (Health Transition scale);^34^ modified patient enablement scale (as developed for the ATEAM trial);^16,35^ and information regarding current or recent medication and treatment. Participants also completed a short weekly questionnaire before each lesson comprising: NRS, modified RMDQ, days in pain, days interference. The NRS and RMDQ were provisionally chosen as core outcomes based on the Core Outcome Measures in Effectiveness Trials (COMET) initiative.^36^ Days in pain and days interference in normal activities were chosen in addition to the RMDQ as these were all used in the ATEAM and Alexander Technique and Supervised Physiotherapy Exercises in back paIN (ASPEN) studies of AT^16,37^.

### Methods of analysis

#### Qualitative analysis

The transcripts were coded and analysed using inductive thematic analysis.^38^ The transcripts were read and re-read. Through initial coding, an early coding frame was developed and discussed in detail by JL and AG. Following agreement, the rest of the data were coded. From these codes, higher order themes were developed, drawing on frequent discussion. When themes had been developed, they were discussed and agreed with the full research team.

#### Quantitative analysis

Baseline and 12-week follow-up scores for outcome measures, and weekly session scores where available, were analysed descriptively using means and standard deviations.

### Patient and public involvement and engagement input

Two patient and public involvement and engagement (PPIE) collaborators provided input to the initial development of the intervention, the protocol, patient and recruitment materials, and study documents. Both PPIE collaborators unfortunately fell ill near the beginning of the development of the intervention and were not able to contribute further to the study.

## Results

Four general practices recruited participants between 8 April 2022 and 18 October 2022. In total, 1375 invitations were sent with 154 replies:

1. Interested and eligible on the NRS: **62**
2. Interested but not eligible on the NRS: **2**
3. Not interested: **90**

In total, 52 patients were screened; 32 were eligible and 25 agreed to participate and signed consent forms.

**Figure 1.**
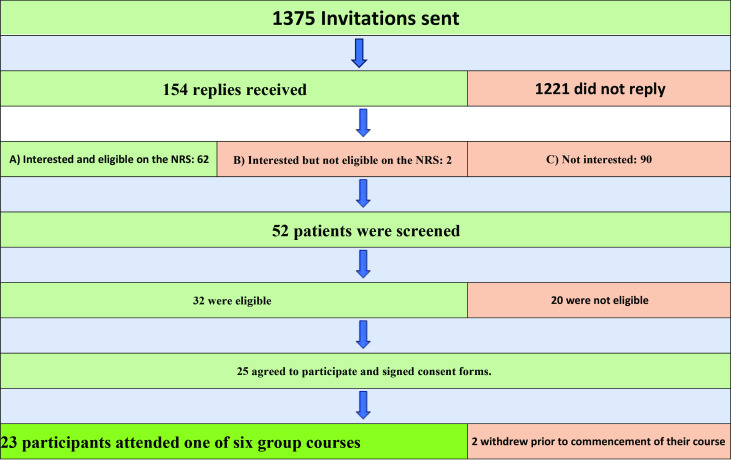
Study flow diagram. NRS = Numerical Rating Scale

Of those 25, two withdrew before starting their group course; the remaining 23 participants attended one of six group courses between 13 July 2022 and 9 March 2023, and 18 agreed to an interview (see study flow diagram Figure 1 and Table 1).

**Table 1.** Baseline sample demographics (*n* = 23)

Age, years		Sex		Ethnicity		Marital status		Age finished continuous full-time education, years		Current employment status	
	*n* (%)		*n* (%)		*n* (%)		*n* (%)		*n* (%)		*n* (%)
20–29	1 (4%)	Male	2 (9%)	White British	22 (96%)	Single	3 (13%)	16 or 17	8 (35%)	Full-time employee	9 (39%)
30–39	3 (13%)	Female	21 (91%)	Asian British	1 (4%)	Married	14 (61%)	18 or 19	3 (13%)	Part-time employee	4 (17%)
40–49	3 (13%)					Living with partner	2 (9%)	20–23	7 (30%)	Retired	3 (13%)
60–69	9 (39%)					Divorced	2 (9%)	≥24	3 (13%)	Homemaker	7 (30%)
70–79	6 (26%)					Widowed	2 (9%)	Not stated:	2 (9%)		
80–89	1 (4%)							Mean:	20 years		

### Qualitative findings

A total of 18 participants agreed to be interviewed as part of the study. Most interviewees were female (*n* = 16/18), in employment (*n* = 9/18), White British (*n* = 17/18), married or living with a partner (*n* = 13/18), and had a range of educational levels. Ages of interviewees varied from 33 years to 81 years and the mean age of interviewees was 65 years old (see Table 2).

**Table 2.** Interview sample demographics (*n* = 18)

Age, years		Sex		Ethnicity		Marital status		Age finished continuous full-time education, years		Current employment status	
	*n* (%)		*n* (%)		*n* (%)		*n* (%)		*n* (%)		*n* (%)
30–39	1 (6%)	Male	2 (11%)	White British	17 (94%)	Single	1 (6%)	16 or 17	7 (39%)	Full-time employee	6 (33%)
40–49	2 (11%)	Female	16 (89%)	Asian British	1 (6%)	Married	12 (67%)	18 or 19	2 (11%)	Part-time employee	3 (17%)
60–69	8 (44%)					Living with partner	1 (6%)	20–23	6 (33%)	Retired	2 (11%)
70–79	6 (33%)					Divorced	2 (11%)	≥24	1 (6%)	Homemaker	7 (39%)
80–89	1 (6%)					Widowed	2 (11%)	Not stated	2 (11%)		
Mean:	65 years							Mean:	20 years		

We developed five central themes regarding attitudes to group AT teaching. Of these, four relate to the effect of including participants with a mix of different musculoskeletal pain sites in AT group lessons, and one to the experience of learning AT in group lessons.

#### Different problems, different results

Of those participants with multiple pain sites, some felt that their learning and practice of AT had variable success in addressing their issues from one pain site to another. For some, this was merely different extent of benefit; for example, one pain site was felt to be considerably improved while another site was not affected whatsoever. There was no discernible pattern with regard to neck, hip, or back pain being reliably less influenced by the application of AT. Participants with knee pain, who perceived that there was underlying structural damage, expressed the view that the technique could not ameliorate the pain by changing body use alone:

*'So it didn't — I didn't think it helped my lower back but it really helped with my upper back and neck and shoulders quite a lot, so — and I'm still — I've taken a bit of a break after stopping *[the course] *and then I've started doing the Alexander Technique again because it does help.'* (GRACE008)

*'Erm, I think, erm, certainly with the neck, erm, neck and shoulder, that, that has, erm, that has helped. Erm, er, perhaps not so much with, erm hip and knee … and, as I say, I know, I know the, the knee, I did injure it, erm, some years ago and I've had two arthroscopies. So know that that is a bit vulnerable, you know, so I, erm, I kind of protect it …'* (GRACE010)

*'Oh, difficult. Erm, certain-, I, er - the, the (low) back pain is a lot more, lot more, erm, easy to manage. When I get — start getting twinges, erm, and bits, bits, yeah, sure, I still do the, sort of, stretching exercises, erm, that, you know, that pull out the sciatic nerve but I also do the Alexander Techniques* [chuckles] *of, of, of, of pushing, you know, relaxing the back and putting meself into good posture positions and so on and so forth. I've got a — the bad knee is, is not as responsive because I think it’s basically damaged arthritic cartilage, damaged and that sort of thing, you know …'* (GRACE011)

#### Whole-body technique, whole-body benefit

In contrast to the above, a common view expressed by participants with multiple pain sites was that the technique had addressed their various issues to a similar degree. Those who expressed this view often related this to their perception of AT as being a whole body or even more broadly holistic approach and therefore whole-body benefits were unsurprising:

*'Alexander Technique has made me think differently, which I think is a fundamental change ... it is the awareness, well, becoming more aware of how your body responds in day-to-day tasks, that you don't actually realise you are screwing yourself up like a ball in various parts of your body, and causing yourself pain or increased sensitivity to pain.'* (GRACE009)

*'I feel it, I feel it’s holistic and I feel that it, it’s, it’s, it helps my mood as well as, as physically … Well, I feel it isn't just a, a physical, erm, activity, but it is actually having an effect upon my, erm, my whole body, but also having an effect on my mind and my spirit.'* (GRACE004)

*'Erm, it was just interesting, and it made you think, and it made you more aware of your own body and your own difficulties and how you did. So it added to it. Because, you know, my needs are so specific, and I must admit, I thought to myself, "I don't think you're gonna really help me." However, after the first lesson, I nearly hugged — er, I can't even remember her name now, whatever she was called, er, because I thought, "Ah, I see. I think — I think this is gonna help."'* (GRACE013)

#### Mix of musculoskeletal problems enhanced learning

Many participants cited the different problems people came to the group lessons with as enhancing their learning in one form or another. In some cases, the opportunity to observe others practising AT allowed participants to develop a better understanding of the technique itself. But also, the sharing of diverse experiences and struggles encouraged a sense of group solidarity, which participants felt supported and motivated by.

*'Well, I, I think it helped, helped the learning process, and also the encouragement, because we're all, we're all at different stages of what we could do obviously, being — you know, having just different physical issues; but it was, um, yes, I, I enjoyed the group, the group sessions.'* (GRACE003)

*'Yeah. I'm sort of trying to think, 'cause I'm thinking back and I'm like, well, what did I learn more. I think I probably, I suppose I learned more about the AT technique and the sort of the science behind it, and seeing it applied on lots of different people in the group sessions.'* (GRACE001)

*'Yes, I found them very useful, and to share sort of experiences with other people, er, was very helpful, and it might not have been what you were experiencing but we, we were able to, I think just support each other.'* (GRACE006)

#### Mix of musculoskeletal problems as a barrier to learning

Although participants did not cite the range of musculoskeletal problems present in the group itself as a barrier to learning, some did express a preference for the kind of specific and detailed personal assessment of their problems offered by individual lessons:

*'Um, yeah, I do, because in the individual lessons it was sort of more tailored to me and my body, and my conditions, and stuff, whereas with the group it was more generalised. I think I preferred the individual stuff because of that, it was more tailored kind of experience of it.'* (GRACE14)

*'Er, no, only that really because a-, at that point someone is looking at you, and only you, and seeing what is wrong, and sort of, um, er, well, just stressing you know, how to cope with it really.'* (GRACE17)

#### Preferences for individual or group lessons

Some participants preferred the individual lesson format, usually citing the teacher’s ability to focus on their specific problems. Others preferred group lessons, usually citing the value of sharing experiences with others in the group and the sense of group solidarity they experienced. Overall, most participants expressed the view that both group and individual lessons contributed something to their learning, and each was valuable in its own way, to a greater or lesser degree:

*'No, I think that worked out because the individual sessions — obviously you have the advantage that you've got the very hands-on kind of experience where … they focus on, on me, you know, so — which was helpful, you know. That, that is helpful. Erm, erm, but it was also nice to have that shared experience as well, so I think yeah, two and six and then two, or some other similar type of arrangement. I think that did work*.' (GRACE008)

#### Benefit of learning AT not reflected by outcome measures

Several participants expressed views about the benefit of learning AT that were not adequately captured by the standard questionnaire outcomes used. In particular, this was identified with regard to improved recovery from pain episodes, ability to manage pain, improved confidence, and improved energy levels as a result of AT practice:

'*I'd say the main thing was confidence and understanding what I should and cou-, can't be — I can be doing with own, you know, with my physical state. But I think it’s definitely — it — more in the mind thing, and it’s a confidence of doing — of doing more, and that is just getting out and about and doing what was normal to me*.' (GRACE013)

*'I found it, err — and it didn't help get rid of pain completely, but it certainly helped me know how to alleviate — you know, have moments of trying to alleviate it, and, and to have some, um, amelioration of kind of what I was experiencing*.' (GRACE012)

### Summary of exploratory quantitative findings

The NRS score fell from 5.15 to 3.85 a change of -1.25 by 12 weeks (see Supplementary Figure 1 and Supplementary Table 1). The modified RMDQ score fell from 8.26 to 5.70 a change of -2.56 by 12 weeks (Supplementary Figure 2 and Supplementary Table 1). Excluding one outlier with a very severe flare of pain at the end did not alter the results meaningfully, the NRS falling from 5.11 (0.38) to 3.75 (0.52) and the RMDQ 8.55 (1.1) to 5.36 (1.23).

Overall, mean days in pain and days interference in normal activities during the last week fell by 0.59 and 1.13, respectively, with consistent weekly downward trends (Supplementary Figures 3 and 4).

Mean modified enablement scores increased substantially (on average at the end of the course nearly all participants agreeing that they were enabled to manage their pain), which matches the qualitative findings, and with similar substantial changes in mean global improvement.

### Preliminary teacher feedback

Feedback following using the course from the teachers who ran the course was positive but some commented that the curriculum needed further development to allow for more flexible presentations by individual AT teachers in order to suit the requirements of their specific group.

## Discussion

### Summary

This study reports the development and implementation of a course of combined group and individual lessons in AT for neck, hip, and knee pain. Overall, the course was acceptable, with perception of important benefits from most participants, but less so among those who perceived they had structural knee problems.

### Strengths and limitations

A strength of this study was the development of this intervention building on the feedback from teachers and participants in the earlier GREAT feasibility study.^30^ We have used mixed methods to gain better insight regarding patients’ views regarding acceptability of the intervention and the experience of learning AT in a mixed group and individual format. The mailed invitations to potential participants provided low uptake rates, as would be expected, but of those assessed for eligibility more than 60% were eligible. Other very large pragmatic trials using such ‘cold calling’ invitation^39^ have demonstrated similar behavioural intentions outside the trial context,^40^ which suggests the results of trials using this method of invitation may be generalisable. Although, the participants included a range of ages, employment, and educational levels, there were very few from non-White ethnic groups, and many more females than males, but that in part reflects the population of those with significant OA. The small sample size and preliminary format warrant caution regarding the descriptive quantitative outcomes. The study was also uncontrolled, so we cannot exclude non-specific changes over time, but the evidence from the qualitative work suggests these were not non-specific effects with participants using the AT to learn better how to manage their pain.

The group teaching incorporated in this intervention should not be considered ‘typical’ within the AT profession at present and a number of teachers interviewed remarked on this non-typical approach (both those who taught the course and those who did not). The group sessions included hands-on work and the numbers were kept small enough for this to be manageable for teachers. The groups were also accompanied by required reading and MP3 talks; again, most groups do not do that. Furthermore, the course as a whole was designed by CN and senior colleagues to be effective for people with neck, hip, and knee pain. Although we do not have data on the use of additional resources (for example, use of MP3 talks) it would likely be sensible for future trials to retain these elements.

### Comparison with existing literature

Echoing the findings of the GREAT study qualitative analysis,^30^ the reception of this intervention by patient participants for the most part was very positive, in many cases finding some advantages to individual lessons alone. Furthermore, based on this analysis the inclusion of patients with a mix of musculoskeletal problems does not appear to have obstructed their ability to learn in a group environment — with many participants finding the diversity of conditions included to be helpful — both in that it encouraged the fostering of group solidarity through sharing their different experiences and in that it was sometimes instructive to see how AT was applied to the problems of others.

Some patients found the technique beneficial across their multiple pain sites — as might be expected, given that AT is a whole-body approach — but that was not a universal finding, particularly for those with severe knee pain. Some participants felt that there was too much structural damage for AT to be able to help, which suggests that future trials should consider excluding participants who perceived they have structural problems with their knees.

The exploratory quantitative outcomes collected as part of this feasibility study should be treated with caution, but suggest clinically important improvements could possibly occur in the primary outcome and secondary outcomes. The reduction in NRS of 1.25 would meet the minimum clinically important change of a 15–20% reduction,^41,42^ and the reduction in modified RMDQ (-2.52 by 3 months) would exceed the minimum clinically important change of 2–2.5.^17^ However, these changes are relatively modest, and given almost universal participant report of considerable benefit based on the qualitative data, perhaps other outcomes may be more sensitive in detecting benefit. It should also be noted there were substantial changes in both the enablement score and in global improvement.

### Implications for research and practice

The importance of this study relates to the limited evidence for interventions for some of the most common musculoskeletal conditions: neck, hip, and knee pain. These three sites were chosen based on some prior evidence, but more evidence is needed. This study reports the very preliminary development and acceptability of a course of individual and group lessons for musculoskeletal pain, but it is clearly premature to make recommendations for practice. A full trial is needed but before a full trial is considered, more PPIE input is needed and further exploration of the key outcomes that most matter for patients; outcomes such as perception of global improvement and enablement could potentially be considered. Consideration is needed whether to include participants with knee pain who perceive that there is structural damage to their joints. Similarly, teachers commented that in further development of the course more room could be made to facilitate personalising the course.

## Data

Data is available on request from the corresponding author, PL, with a proposal for further analysis.
